# Traditional Chinese Medicine Bionic Tiger Bone Powder for the Treatment of AI-Associated Musculoskeletal Symptoms 

**DOI:** 10.1155/2017/2478565

**Published:** 2017-01-31

**Authors:** Yifan Li, Zhenhua Zhang, Feifei Cui, Jialing Liu, Yitong Wang, Juling Jiang, Wenxin Ma, Wenping Lu

**Affiliations:** Oncology Department, Guang'anmen Hospital, No. 5, Beixiange, Xi Cheng District, Beijing 100053, China

## Abstract

*Background*. Aromatase inhibitors (AIs) are used for adjuvant therapy of breast cancer; however AIMSS (AI-Associated Musculoskeletal Symptoms) can negatively affect quality of life and compliance. Most patients in China moved to TCM (traditional Chinese medicine) for help. TB (tiger bone) is used to treat bone disease, whose main ingredients are calcium and collagen. The objective of this study was to evaluate whether the TB prevented AIMSS in postmenopausal women with ER/PR+ breast cancer.* Methods*. We conducted a randomized, blind, controlled study of comparing TB versus placebo for 12 weeks in postmenopausal women with breast cancer who have taken AI for less than a month. Patients completed the M-BPI, VAS, and FACT-B at baseline, 6 weeks, and 12 weeks. M-BPI and VAS were used as the primary outcomes. FACT-B was used as the secondary outcome. Serum E_2_ and FSH were tested every 6 weeks.* Results*. Of 70 evaluable cases, 8 of 35 patients (22.9%) developed new or worsening point symptoms in TB group, compared to 21 of 35 (60%) in placebo group (*P* < 0.001). We also found differences between 2 groups in average pain (2 to 5.6), worst pain (3.9 to 8), pain interference severity (1.9 to 5.3), stiffness (2.4 to 6.9), and joint symptom interference (1.8 to 5.7), all *P* < 0.001; similar findings were seen in VAS value (3 to 6.6) at the end of intervention. HRQoL measured by FACT-B (*P* < 0.05) was improved. No change of serum estradiol and FSH between two groups.* Conclusions*. TB appeared to be effective and safe in the prevention of AIMSS. This trial is registered with ChiCTR-IPR-15007081.

## 1. Introduction

It has been proved that AI is more effective in decreasing the risk of recurrence of breast cancer than Tamoxifen [1], which indicates the important role of AI for adjuvant therapy of postmenopausal breast cancer. What is more, the results of MA.17R clinical trials showed that patients received 10 years' AI treatment could get more benefits than those received 5 years' therapy on American Society of Clinical Oncology (ASCO), 2016. Although large clinical trials have proved AIs to be effective and safe [2], follow-up reports showed that more than 50% of the patients had developed AI-Associated Musculoskeletal Symptoms (AIMSS) [3], which include arthralgia and stiffness of hands, knees, hips, shoulders, and back. In a cross-sectional survey of 200 patients receiving adjuvant AI therapy for breast cancer, 94 (47%) reported AI-associated joint pain, and 88 (44%) reported joint stiffness; about 13% of patients discontinued therapy because of musculoskeletal symptoms [4]. Due to the unknown mechanism of AIMSS, common treatments mainly aim at relieving the symptoms, including nonsteroidal anti-inflammatory drugs, analgesics, calcitriol agents, vitamin D, and exercise. However, most patients cannot get satisfying effects [5]. Consequences are that AIMSS not only impairs the quality of patients' life, but also hinders their compliance, which will definitely disrupt further treatments.

Tiger bone (TB) is one precious Chinese medicine material. In traditional Chinese medicine (TCM), tiger bone is used to treat chronic pains by its functions of strengthening the muscles and bones, expelling wind and cold, and relieving pain and convulsion [6]. Given tigers are protected animals, scientists adopted bionic research method to develop bionic tiger bone powder, which has similar ingredients to natural tiger bone. Modern studies have shown prominent anti-inflammatory, analgesic, and antiosteoporotic effects of bionic TB [7]. Hence bionic TB powder has taken place of TB and has been widely used in clinical practice to treat osteoporosis and arthrosis.

Based on that, we speculated that TB might have an active effect on AIMSS and put that into practice. The good news is that we noted many patients with AIMSS could get satisfying relief by bionic TB powder in clinical practice, with rare bad effects. However, the research in this area is limited, so we conducted this randomized controlled double-blind clinical study to evaluate the effect and safety of TB powder to relieve the symptoms of AI-associated arthralgia, so as to provide clinical evidence.

## 2. Materials and Methods

### 2.1. Participants

We recruited eligible patients from Guang'anmen Hospital, China Academy of Chinese Medicine, Beijing. 72 patients were enrolled, of which 70 were evaluable.

Inclusion criteria were the following: (1) pathologically confirmed stage 0 to III breast cancer with no recurrence and metastasis, (2) postmenopausal women including ovarian suppression and natural postmenopause, (3) current use of a third-generation aromatase inhibitor (anastrozole, letrozole, or exemestane) for no more than one month, (4) accompanied by recent symptoms of joint pains, (5) Eastern Cooperative Oncology Group performance status 0–III, and (6) provided written informed consent before enrollment.

Exclusion criteria were the following: (1) patients with endocrine and any other diseases influencing bone metabolism (e.g., hyperthyroidism, hypothyroidism, diabetes, Cushing's syndrome, chronic liver disease, nephropathy, myeloma, bone tumor, or bone metastasis), (2) use of agents influencing bone metabolism (e.g., glucocorticoid, thyroid hormone, heparin, anticonvulsants, diuretics, or bisphosphonates), except calcium agents, within the past 3 months, (3) contraindications to calcium agents and vitamin D, (4) diagnosis of primary osteoarticular diseases, (5) presence of other primary tumors and severe heart, liver, kidney, and hematopoietic system diseases, and (6) presence of pregnancy, mental illness, or cognitive handicap.

### 2.2. Interventions

72 patients were randomly assigned to bionic tiger bone powder or placebo group, 36 patients in each group. A randomization list was prepared using random permuted blocks, and consecutive assignments were placed into separate numbered, sealed envelopes. And group assignments will not be revealed until the entire study is completed. Participants, investigators, statisticians, and all study staff are blinded, under the supervision of Ethics Committee of Guang'anmen Hospital.

Participants in TB group were given tiger bone powder made into capsules, which have been approved by China Food and Drug Administration (CFDA: Z20030080) to treat arthralgia, and the recommended dose is 1.2 g, 3 times daily. Participants in placebo group were given calcium carbonate tablets (CFDA: H10950029), whose main ingredients are calcium and vitamin D, with recommended dose of 600 mg daily. Both medicines were asked to be taken with an interval of more than half an hour from AI and lasted 12 weeks.

### 2.3. Outcome Measurements

Clinical information was obtained via chart abstraction. Patients were asked to complete the following questionnaires at baseline, week 6, and week 12 to evaluate change in pain and functional status.

Modified Brief Pain Inventory (M-BPI) and pain Visual Analog Scale (VAS) were adopted to evaluate the pain and functional status. The M-BPI is one of the most widely used instruments to measure pain in cancer patients and has been demonstrated to be a reliable, valid, and responsive measure [8]. The numerical rating scale ranges from 0 to 10, with 10 indicating the greatest severity or interference. We modified the inventory by adding “stiffness” (one of the most common symptoms of AIMSS) and “joint symptom interference” to quantify joint stiffness status and evaluate the pain more specifically, on the same 0–10 scale. Joint symptom incidence rate and severity of the pain including average pain, worst pain, pain interference severity, stiffness, and joint symptom interference by M-BPI and VAS were used as the primary outcomes.

Health-Related Quality of Life (HRQoL) was measured by the Functional Assessment of Cancer Therapy-Breast (FACT-B). FACT-B measures physical, social/family, emotional, and functional well-being and additional concerns [9]. The FACT-B scale has 5 response levels (“not at all” to “very much”), where higher scores reflect better well-being and fewer symptoms. FACT-B was used as the secondary outcome.

Considering that breast cancer is a hormone-related disease, we also assayed estradiol (E_2_) and follicle-stimulating hormone (FSH) from patients' serum samples at the baseline, week 6, and week 12 time point, so as to see if the intervention would influence patients' hormone levels.

### 2.4. Statistical Analysis

We described demographic, clinical, and outcome variables by using means and standard deviations for continuous variables and percentages for categoric variables by treatment group. Data analysis was performed using the Statistical Packages of Social Sciences software (SPSS 20.0). Statistical significance will be defined as two-sided *P* value of <0.05. The outcome comparison between the 2 groups was compared using paired* t*-test.

## 3. Results and Discussion

### 3.1. Participant Characteristics

Between May 2015 and May 2016, 72 participants were enrolled and completed baseline questionnaires; 70 of them were evaluable. One patient immigrated to Japan, and another patient had scheduling difficulties to continue. All evaluable patients were randomly assigned. The baseline characteristics of the unevaluable participants were not significantly different from the treated groups.

Baseline demographics and clinical characteristics were comparable between the two groups (Table 1). The median age of the women enrolled was 57 years (range, 27 to 73 years). More than half of the participants were naturally menopausal. And more than half of the patients took anastrozole instead of letrozole and exemestane. Most of them were diagnosed I to II pathological stage.

### 3.2. Reduction of Pain

Of the 35 patients in TB group, 8 of them (22.9%) developed new or worsening joint symptoms, compared with 21 of 35 (60%) in placebo (*P* < 0.001). The mean pain score at baseline, as measured by the M-BPI average pain item on a scale of 0 to 10, was 4.6 and 4.9 for the TB and placebo, respectively (Table 2). At 12 weeks, the mean M-BPI average pain score was 2.0 and 5.6 (*P* < 0.001) for the TB and placebo groups, respectively, corresponding to 50% improvement in scores compared with baseline in the TB group. Similar findings were seen for worst pain (3.9 versus 8.0), pain interference severity (1.9 versus 5.3), stiffness (2.4 versus 6.9), and joint symptom interference (1.8 versus 5.7), all *P* < 0.001. There were 26 patients reporting over 2 points' decrease for average pain in TB group (74.3%) at 12 weeks. And 31 patients reported stiffness at baseline in TB group, and 27 of them reported over 2 points' decrease (87.1%).

Similar findings were seen for VAS value at the end of intervention. At baseline, mean pain value was 6.3 and 6.4 (*P* = 0.874) for the TB and placebo groups, respectively. And at 12 weeks, the scores were 3.0 and 6.6 (*P* < 0.001) for the TB and placebo groups, respectively, corresponding to 50% improvement in scores compared with baseline for the TB group (*P* < 0.05). Of 35 participants in TB group, 20 reported over 2 points' decrease (57.1%).

### 3.3. Health-Related Quality of Life (HRQoL)

HRQoL measured by FACT-B was improved. Physical well-being measured by the FACT-B showed a significant improvement for TB group compared with placebo group (22.23 versus 19.93; *P* < 0.05) (Table 3). And TB group showed a significant improvement at physical well-being on week 12 (*P* < 0.05). No significant differences were observed for the FACT-B social/family, emotional, and functional well-being subscales and additional concerns.

### 3.4. Influences on Serum Hormones

No significant differences were found for the estradiol (E_2_) and follicle-stimulating hormone (FSH) on 6 weeks or 12 weeks in either group (all *P* > 0.05), and there were no significant differences between the two groups in 12 weeks' time (*P* > 0.05) (Table 4).

### 3.5. Safety and Tolerability

Of all 72 enrolled participants, 6 of them (2 in TB and 4 in placebo) reported stomach discomfort, but tolerable. So we suggested to them taking pills half an hour after meals, and no other adverse events were reported. And 2 participants failed to continue the intervention. One in TB group lost her mother at 3 weeks, so that she had difficulties in scheduling. Another participant in placebo group, her husband had a job change, and the whole family had moved to Japan on week 8. Other 70 participants had finished the intervention, and there were no lost cases.

### 3.6. Consort Diagram

See Figure 1.

## 4. Discussion

AIs have been shown to cause arthralgia in up to 46% of patients [10], which can be difficult to treat and can negatively impact quality of life and become potentially persistent with therapy, which makes it obliged to look for effective treatments [5, 11, 12]. However, the exact pathogenesis of AIMSS stays unknown, and present therapies mainly aim at relieving symptoms, including nonsteroidal anti-inflammatory drugs, analgesics, calcitriol agents, vitamin D, and exercise, whereas the effects are not as much satisfying, given that doctors keep searching for more effective complementary and alternative therapies. A number of small interventional trials investigating vitamin D [13], duloxetine [14], acupuncture [15–17], electroacupuncture [18], glucosamine [19], prednisolone [20], yoga [21], and TCM formulas [22–24] have provided various treatment methods. Compared with aforesaid trials, this research has a relatively larger sample size, with randomized controlled double-blind design, presenting us a more powerful evidence to support the TB therapy. Besides, compared with other interventions, such as acupuncture, electroacupuncture, and yoga, TB therapy is painless, and it is easier to handle for there is no requirement of doctors' specific skills. Also, compared with some traditional Chinese medicine formulas, which usually consist of various herbs and complicated ingredients, TB alone is simpler, making it easier to study the mechanism of the effect and safety.

Speaking of AIMSS, there is not specific record of it in traditional Chinese medicine literature, but it should be attributed to bone rheumatism. That is mainly because postmenopausal women with AIMSS usually exhibit a specific pathological change associated with deficiency of kidney essence, such as pain, stiffness, and dysfunction [25]. Chinese traditional medicine theory considers that kidneys are generating essence and governing bones. And the major pathogenesis of bone rheumatism is that the deficiency of kidney essence results in failure to nourish the related body constituents and organs, especially bone and joints; thus pathogenic factors such as wind and cold would take advantage of the deficiency to “hit” body. Based on that, treatments should focus on tonifying the kidney, fortifying the bone, and dispelling the pathogenic factors. Meanwhile, records in* Compendium of Materia Medica* say that TB has the function of strengthening bone, expelling wind, and alleviating pain. Those theories drove us to seek whether TB could treat AIMSS and to explore its mechanism, potential efficacy, and safety.

TB is commonly used to treat musculoskeletal symptoms, including bone pain, myalgia, and arthralgia, in patients with osteoporosis or arthrosis [6]. Developed by bionic research method, bionic tiger bone powder shows no significant differences with natural tiger bone in ingredients and pharmacologic effects. Modern pharmacological research has shown that bionic TB powder has more than 20 kinds of amino-acid and microelements essential to human. Besides, calcium to phosphorus ratio of TB makes it suitable for body to absorb, whereas it also contains various organic components, such as collagen, analgesic peptide, bone morphogenetic protein, bone growth factors, and polyose. Bionic TB powder shows the functions of anti-inflammation, abirritation, sedation, healing fracture, and improving bone strength [26, 27]. Those above may be the mechanism of TB treating AIMSS.

In this study, we confirmed that postmenopausal women with AI-induced arthralgia demonstrate an improvement in joint pain, stiffness, and functional ability after 12 weeks of TB therapy. 74.3% participants completed TB therapy experienced at least a 2-point decrease in patient-reported average pain, and 84.1% reported at least a 2-point decrease in stiffness. This is an improvement that has been shown to be clinically meaningful in studies of other chronic pain conditions and that is the cut-off recommended by the Initiative on Methods, Measurement, and Pain Assessment in Clinical Trials consensus committee [28]. No significant benefits were observed in women randomly assigned to placebo group.

As for HRQoL measured by FACT-B, the improvement of physical well-being had statistical significance; however no same results were seen for other items. That might be mainly due to the impact on pain of TB, and the pain was improved, thus influencing participants' physical well-being, which indicates that TB has no significant effect on other parts of life for breast cancer patients treated with AIs.

AIs treat breast cancer mainly by reducing estrogen conversion, thus lowering serum estradiol level [29]. In this study, no significant differences were found in serum E_2_ and FSH change after 12 weeks' intervention, both in 2 groups, which proved that TB had no impact on AIs' curative effect on breast cancer, ensuring the safety of TB treating AIMSS. In addition, the TB intervention was well-tolerated, with minimal side effects.

There are still many deficiencies due to our limitations that should be focused on in further study. Larger number of participants should be enrolled so that the result would be more convincing. Besides, more biomarkers should be conducted to evaluate the response to TB therapy such as inflammatory biomarkers. Besides, longer-term studies are necessary to confirm the durability of the response. In addition, we should try to build multicenters to process the following study.

Above all, with more evidence proving the benefit of long-term AI therapy in the adjuvant setting, AI-induced arthralgia is becoming one of major issues for breast cancer survivors. However, given the profound benefits associated with these medications, the majority of patients suffering from AIMSS choose to continue AI therapy. Our study indicates that TB can play an important role in assisting traditional Chinese herbal medicine in the long-term treatment for AIMSS, in order to alleviate adverse reaction and improve the quality of life much better.

## 5. Conclusions

In conclusion, these results strongly suggest that TB is effective and safe in decreasing AIMSS of hormone receptor-positive breast cancer women. Given the high incidence of AIMSS, identification of therapy to ameliorate these treatment emergent toxicities is important to optimize persistence with therapy and quality of life.

## Figures and Tables

**Figure 1:**
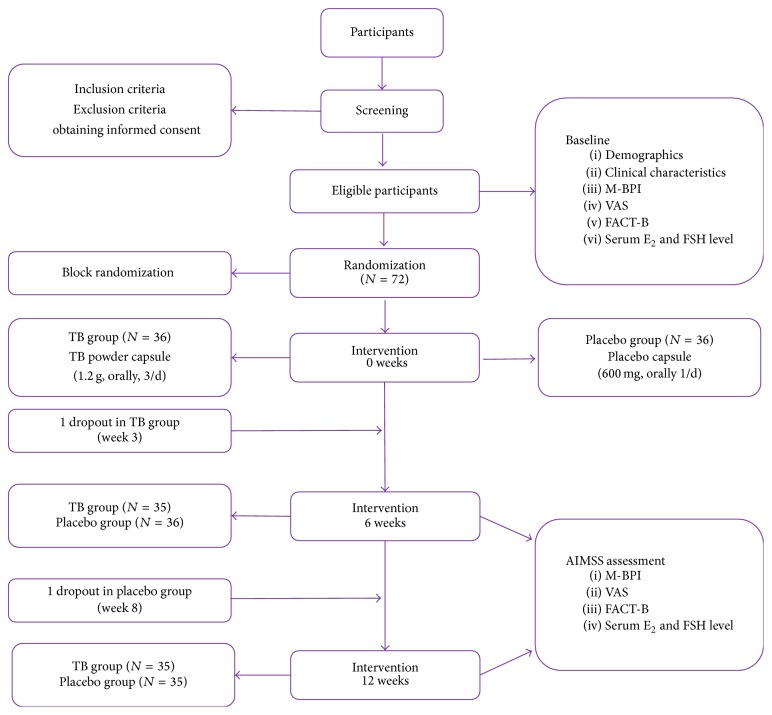


**Table 1:** Baseline demographics and clinical characteristics.

Characteristic	TB (*n* = 35)	Placebo (*n* = 35)
Age, years		
Median	55	52
Range	27–73	31–72
Body mass index		
Median	27.6	26.7
Range	17.5–36.8	18.1–35.9
Menopause		
Chemically induced (%)	12 (34.3%)	11 (31.4%)
Surgically induced (%)	4 (11.4%)	2 (5.7%)
Natural (%)	19 (54.3%)	22 (62.9%)
Years since menopause		
Median	9	10
Range	0.1–28	0.1–26
Aromatase inhibitor therapy		
Anastrozole	21 (60.0%)	18 (51.4%)
Letrozole	10 (28.6%)	11 (31.4%)
Exemestane	4 (11.4%)	6 (17.1%)
Pathological state		
0	2 (5.7%)	1 (2.9%)
I	18 (51.4%)	16 (45.7%)
II	14 (40.0%)	15 (42.9%)
III	1 (2.9%)	3 (8.6%)
Adjuvant chemotherapy	25 (71.4%)	21 (60.0%)
Adjuvant taxane	13 (37.1%)	8 (22.9%)

**Table 2:** Comparison of M-BPI and VAS outcomes.

Outcome measure and time point	TB (*n* = 35)		Placebo (*n* = 35)		*P* value
	Mean	SD	Mean	SD	
M-BPI					
Average pain (0–10)					
Baseline	4.61	2.31	4.89	2.27	
6 weeks	3.09	2.08	5.76	2.19	
12 weeks	1.99 ☆	1.79	5.64	2.17	△
Worst pain (0–10)					
Baseline	6.59	2.11	7.09	2.43	
6 weeks	5.42	2.07	7.53	2.18	
12 weeks	3.87	2.56	7.96	2.39	△
Pain interference severity (0–10)					
Baseline	4.14	1.79	4.39	1.52	
6 weeks	2.94	2.46	4.78	2.53	
12 weeks	1.91 ☆	1.95	5.28	2.04	△
Stiffness (0–10)					
Baseline	5.49	1.58	5.07	2.74	
6 weeks	4.33	1.73	5.78	2.18	
12 weeks	2.41 ☆	2.36	6.94	2.59	△
Joint symptom interference (0–10)					
Baseline	3.19	2.43	2.90	1.58	
6 weeks	2.60	2.48	3.79	2.15	
12 weeks	1.80 ◯	1.65	5.70	1.57	△
VAS (0–10)					
Baseline	6.34	1.68	6.41	2.07	
6 weeks	4.52	2.42	6.95	1.98	
12 weeks	3.03 ☆	1.56	6.62	1.77	△

△ represents *P* values based on comparison between two groups at 12 weeks, and *P* < 0.001

☆ represents *P* values based on comparison of baseline in one group, and *P* < 0.001

◯ represents *P* values based on comparison of baseline in one group, and *P* < 0.05.

**Table 3:** Comparison of FACT-B outcomes.

Outcome measure and time point	TB (*n* = 35)		Placebo (*n* = 35)		*P* value
	Mean	SD	Mean	SD	
Physical well-being (0–28)					
Baseline	19.23	5.02	20.03	4.82	
6 weeks	20.64	5.63	20.53	3.44	
12 weeks	22.23 ○	4.31	19.93	5.24	□
Social/family well-being (0–28)					
Baseline	19.81	3.98	20.45	5.29	
6 weeks	20.38	4.32	18.97	5.21	
12 weeks	20.74	5.30	19.48	4.32	
Emotional well-being (0–24)					
Baseline	15.34	4.41	14.90	5.68	
6 weeks	15.78	4.29	14.87	6.09	
12 weeks	15.83	4.76	15.02	5.75	
Functional well-being (0–28)					
Baseline	15.26	4.67	15.96	4.09	
6 weeks	16.20	5.77	16.09	5.48	
12 weeks	15.97	5.77	15.87	4.67	
Additional concerns (0–36)					
Baseline	26.73	4.61	27.11	5.22	
6 weeks	27.24	3.98	27.59	4.39	
12 weeks	27.46	5.39	27.86	5.19	

□ represents *P* values based on comparison between two groups at 12 weeks, and *P* < 0.05

○ represents *P* values based on comparison with baseline in one group, and *P* < 0.05.

**Table 4:** Comparison of serum E_2_ and FSH level.

Outcome measure and time point	TB (*n* = 35)		Placebo (*n* = 35)	
	Mean	SD	Mean	SD
E_2_ (pg/mL)				
Baseline	14.24	6.34	13.28	6.46
6 weeks	14.52	7.01	13.44	5.96
12 weeks	14.39	6.58	13.29	6.33
FSH (mIU/mL)				
Baseline	43.73	14.35	44.23	15.54
6 weeks	43.39	15.67	44.19	15.66
12 weeks	43.55	14.52	44.25	15.86
